# Mononeuritis Multiplex Due to Hansen’s Disease: A Look Through Ultrasound and Electrodiagnosis

**DOI:** 10.7759/cureus.44175

**Published:** 2023-08-26

**Authors:** Wilmer Santiago Herrera Malpica, Paula Vanessa Muñetones Hernández, Nathalia Maria Perez Becerra, Jorge Nicolas Muñoz Rodriguez, Jorge Arturo Diaz Ruiz

**Affiliations:** 1 Rehabilitation Medicine, Universidad Nacional De Colombia, Bogotá, COL; 2 General Medicine, Universidad Nacional De Colombia, Bogotá, COL

**Keywords:** nerve conduction studies (ncs), ultrasonography, mononeuritis multiplex, ulnar nerve, hansen's disease

## Abstract

Hansen’s disease is caused by Mycobacterium leprae. This bacillus can invade the peripheral nerves asymmetrically, including the ulnar, median, and radial nerves, causing mononeuritis multiplex. We present the case of a 41-year-old man with a history of Hansen’s disease with sensory and motor symptoms. Electrodiagnostic studies and ultrasound showed asymmetric lesions of the median, ulnar, and radial nerves. Because this is the main complication of this pathology, electrodiagnosis is clearly valuable for its diagnosis, demonstrating axonal and myelin involvement, as well as signs of denervation and reinnervation. Ultrasound is valuable in the detection, diagnosis, and assessment of the extent of mononeuritis multiplex due to Hansen’s disease. It aids in identifying significant inflammatory deterioration, as indicated by increased blood flow in the nerves and enlargement of the nerves. This technique allows for the exploration of nerves such as the ulnar nerve and branches of the brachial plexus. In a complementary way, ultrasound provides information on the severity of the disease. Early diagnosis of this entity is essential because it can generate aesthetic and functional permanent affectation.

## Introduction

Leprosy is a chronic granulomatous disease caused by infection with Mycobacterium leprae (M. leprae). It exhibits a strong affinity for the skin and peripheral nerves, resulting in asymmetric invasion and the development of leprotic neuropathy. This neuropathy causes autonomic, sensory, and motor damage to the peripheral nervous system fibers, leading to physical deformities [1,2]. The clinical response to leprosy depends on the host’s immune response quality [2,3]. It is primarily driven by an immunological hypersensitivity response characterized by the overexpression of proinflammatory cytokines and the formation of immune complexes that damage Schwann cells [1].

M. leprae enters peripheral nerves asymmetrically, crossing the neurovascular endothelium and infiltrating the perineural sheath. It reaches the endoneural compartment, affecting both myelinated and unmyelinated sensory fibers [1], leading to leprotic neuropathy. Neural pathology becomes evident when more than 30% of type C sensory fibers are compromised [1]. Mononeuritis multiplex is the most common presentation associated with leprotic neuropathy (79%), predominantly affecting the upper extremities and involving the ulnar, median, and superficial radial nerves. In cases affecting the lower extremities, it often damages the common peroneal and posterior tibial nerves [2].

Mononeuritis multiplex is characterized by asymmetric, asynchronous, and painful involvement of at least two noncontiguous nerves, resulting in sensory and motor neuropathy. It can be caused by metabolic diseases, inflammatory compressions, infections, rheumatological conditions, or neoplasms [2,4]. Leprosy-associated polyneuropathy is characterized by a loss of exteroceptive sensitivity and is more prevalent in men aged 15-30 years [2,4]. The inflammatory process leads to nerve thickening, which can be palpated along its course and is observed in up to 75% of patients [2].

Electrodiagnostic studies show secondary axonal neuropathy with signs of demyelination over the entire passage through the elbow of the ulnar nerve, and needle electromyography can show signs of denervation [2,5]. Peripheral nerve ultrasound is more sensitive than physical examination for detecting the thickening of the nerves. A correlation is found between hyperechogenic and hypervascular images with the histology of lepromatous leprosy [5].

Ultrasound in mononeuritis multiplex due to Hansen’s disease results in significant inflammatory deterioration of nerve function, an increase in blood flow within the nerves, and enlargement of nerve size as sensitive indicators. This affects crucial nerves in clinical examination, such as the ulnar nerve at the elbow, the tibial nerve at the ankle, the fibular nerve at the head of the fibula, and the greater auricular nerve. The diagnostic utility of ultrasound and color Doppler is valuable in detecting, diagnosing, and evaluating the disease's extent.

## Case presentation

A male patient age 50, with a history of Hansen’s disease, treated approximately 10 years ago (drug treatment with oral dapsone 100 mg per day for six months, rifampin 600 mg per day, and clofazimine 300 mg once a month and 50 mg twice a week for 12 months), was consulted for dysesthesias at the level of fingers 4 and 5 of the right hand, associated with the loss of muscle mass in the hypothenar region with decreased strength in the intrinsic muscles of both hands, predominantly on the right. On physical examination, he presented atrophy of the intrinsic muscles of the hand and weakness of the interosseous and lumbrical muscles of the right hand. The patient had difficulty flexing the metacarpophalangeal joints with a predominance of the thumb and difficulty extending the interphalangeal joints, causing claw hand deformity. At a sensitive level, there is evidently autonomous air anesthesia of the ulnar and radial nerve.

**Figure 1:**
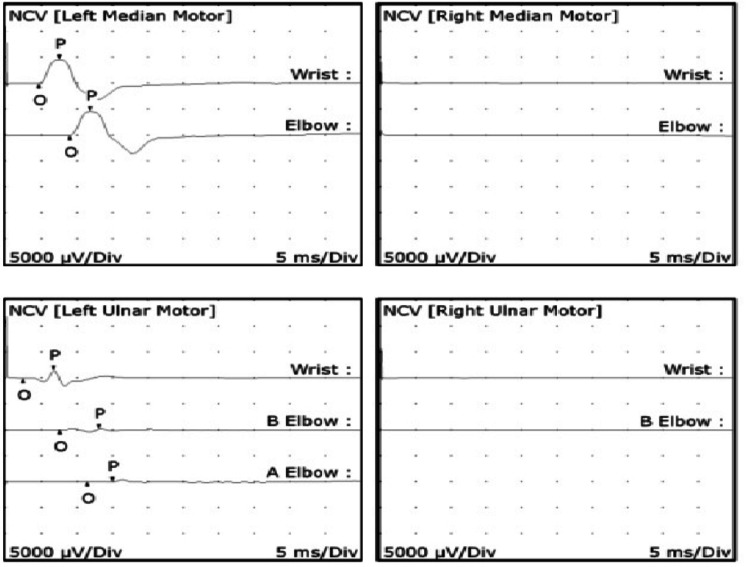
Nerve conduction studies Motor responses of the right median and ulnar nerves were not obtainable. The motor response of the left median nerve showed prolonged latency. The motor responses of the left ulnar nerve have reduced amplitude and were dispersed in shape, with conduction velocity being slow at the elbow level.

**Figure 2:**
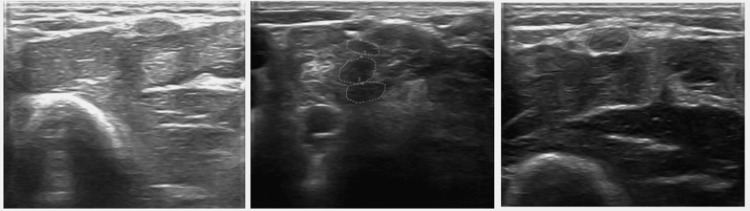
Ultrasound images Ultrasound images of the ulnar nerves using a 15 MHz transducer showing a significant increase in the CSA of the left and right ulnar nerves above the elbow. (Left image) Left ulnar nerve 10 cm above the elbow with a CSA of 16.9 mm ² (center image) thickening of the C5-C6-C7 roots with CSAs of 15.2 mm², 21.5 mm², and 18.7 mm², respectively. (Right image) Right ulnar nerve 10 cm above the elbow with a CSA of 21.6 mm².

He has electrodiagnostic studies (Figure 1) that report the absence of motor response in the median and right ulnar nerves. The patient had a bilateral lack of sensory response in the ulnar, radial, and right median nerves. Needle electromyography in muscles innervated by radial, median, and ulnar nerves showed signs of chronic reinnervation (Table 1). Neuromuscular ultrasonography showed an increase in the cross-sectional area of the ulnar nerve above the elbow of the median nerves at the level of the carpal tunnel and of the C5-C6 and C7 roots (Figure 2). All of the above is compatible with chronic phase asymmetric axonal polyneuropathy (mononeuritis multiplex).

**Table 1:** Needle electromyography findings High-amplitude, long-duration motor unit potentials, along with a reduced recruitment pattern, with signs of chronic reinnervation in the pronator teres, flexor digitorum profundus, and first dorsal interosseous muscles.

Side	Muscle	Nerve	Root	Insertion activity	Fibrillations	Positive sharp waves	Amplitude	Duration	Recruitment
Right	Pronator teres	Median	C6-7	Normal	None	None	Increased	Prolonged	Reduced
Right	Flexor digitorum profundus	Median, 2nd and 3rd digit; ulnar, 4th and 5th	C8-T1	Normal	None	None	Increased	Prolonged	Reduced
Right	First dorsal interosseous muscles	Ulnar	C8-T1	Normal	None	None	Increased	Prolonged	Reduced
Right	Extensor indicis proprius	Radial	C7-8	Normal	None	None	Increased	Prolonged	Reduced
Right	Deltoid	Axillary	C5-6	Normal	None	None	Normal	Normal	Normal
Right	Biceps	Musculocutaneous	C5-6	Normal	None	None	Normal	Normal	Normal

He currently receives medical treatment with a neuromodulator (pregabalin), a nonsteroidal anti-inflammatory in case of pain (naproxen), and performs rubbing therapies for the intrinsic muscles of the hand, flexors, and extensors of the elbow and wrist. He receives occupational therapy for gross and fine motor pattern strategies with follow-up by psychology for psychosocial risk.

## Discussion

M. leprae, a nonmotile acid-fast bacterium, invades Schwann cells, the principal supporting cells in the peripheral nervous system. The direct effect of M. leprae on Schwann cells is not fully understood [6], but impaired nerve function occurs independently of the postinfection immune response. Neuritis, characterized by thickening, pain on palpation, and nerve damage leading to sensory and motor disturbances, is a typical clinical manifestation of leprosy [7].

Leprosy often leads to delayed diagnosis, particularly in cases without classic hypo-/anesthetic dermatological lesions, as seen in pure neural leprosy [8-4]. Leprosy reactions, occurring in 30-50% of leprosy patients, are immune-mediated inflammatory complications associated with both treated and untreated M. leprae infections. These reactions present in two subtypes: type 1 (reversal or degradation) and type 2 reactions (erythema nodosum leprosum). Neuritis is a common manifestation of both reaction types [6]. Biopsies of human lesions have revealed Schwann cell apoptosis, and subsequent inflammatory responses, known as leprosy reactions, are considered to be primarily responsible for the neurological manifestations associated with the bacillus infection [6].

Nerve conduction studies often show more widespread involvement than what is clinically evident. High-resolution ultrasound is an advanced, cost-effective, noninvasive, and rapid method for real-time peripheral nerve assessment. It complements traditional nerve conduction testing by providing information on morphology, size, internal fascicular architecture, and vascularization along the entire nerve length, with good spatial and contrast resolution. Ultrasound provides a more reliable and objective measure of peripheral nerve enlargement in leprosy compared to clinical assessment [8].

In M. leprae infection, the most common nerve lesions are mononeuritis, mononeuritis multiplex, and polyneuritis. They can present with severe neuropathic pain (due to damage to small fibers) that can occur years after skin lesions. It is common to find patients with symptoms of mononeuritis multiplex or mononeuropathy with thickening of the nerve and negative skin biopsy [3-5].

In nerve conduction studies, signs of axonal compromise and demyelination can be found, predominantly in the ulnar and median nerve, due to the absence of both motor and sensory response, including the sensory response of the radial nerve. Regarding electromyography, although signs of active denervation are usually found, in our case, large motor units compatible with symptoms of chronic reinnervation were isolated, which may be secondary to the time of evolution and treatments performed. Similarly, the studies support the approach with neuromuscular ultrasound to complement and diagnose the thickening of the nerves, with an increase in the cross-sectional area (CSA) of the C5, C6, and C7 roots; ulnar nerves above the elbow; and median to carpal tunnel level.

Ultrasonography plays a pivotal role due to its ability to reveal echographic patterns, such as heightened CSA, along with variations in fascicular structure and echogenicity (specifically hypoechogenicity) [5]. This positions ultrasonography as a crucial tool for the early identification of changes in the anatomical framework of peripheral nerves, ultimately contributing to the development of symptoms and functional modifications over time. The identification of abnormal color Doppler signals within nerve fascicles or the epineurium is indicative of irregularities, and the presence of endoneural flow has been observed in patients with active neuritis and reversal reactions in leprosy cases [9].

To locate the roots of the brachial plexus in the oblique sagittal plane, the probe is positioned at the lower third of the sternocleidomastoid muscle, perpendicular to the main axis of the supraclavicular vessels (Figure 2). This positioning reveals the roots as hypoechoic ovoid images nestled between the anterior and middle scalene muscles, thereby providing a reference point for identifying the roots in the oblique sagittal plane: C5, C6, and C7 [10].

Enlarged nerves are a common finding in patients with prior untreated inflammatory neuropathies, leprotic neuropathy, chronic inflammatory demyelinating neuropathy, multifocal motor neuropathy, and Lewis-Sumner syndrome. Thickening of the brachial plexus supports the diagnosis of chronic inflammatory neuropathy. The enlargement of the upper, middle, or lower trunk within the interscalene triangle is defined as a cross-sectional area greater than 8 mm². The sensitivity and specificity of ultrasound can be enhanced by combining assessment of the brachial plexus with peripheral nerves. For instance, a median nerve in the forearm greater than 10 mm², ulnar nerve in the elbow greater than 11 mm² or in the upper arm greater than 13 mm², and any trunk of the brachial plexus larger than 8 mm² [11].

The mononeuritis multiplex due to Hansen’s disease in the patient has led to both aesthetic and functional alterations in the hand. These manifestations include difficulties in grasping objects, reduced grip strength, disruptions in fine motor skills, and sensory impairment that affects the territory of the ulnar and radial nerves. The claw-like deformity in the hand, resulting from muscular atrophy due to denervation, significantly limits the patient's ability to engage in activities and participate fully.

## Conclusions

Hansen’s disease (leprosy) still faces social stigma, but significant progress has been made in its understanding and treatment. Psychological support and follow-up are crucial for patients affected by the disease’s devastating impact on the peripheral nerves. Without early diagnosis, leprosy can lead to aesthetic and functional impairments due to sensory and motor alterations. Ultrasonography serves as a valuable tool for the early detection of anomalies, showcasing echographic patterns such as increased CSA, modifications in fascicular structure, and changes in echogenicity. These alterations can lead to symptoms and functional changes over time. As a supplementary diagnostic approach, ultrasonography highlights asymmetrical nerve thickening with hypogenicity and fascicular hypertrophy. The effectiveness of high-resolution bidimensional and Doppler ultrasonography enables the measurement of CSA, assessment of epineurium diameter, and identification of abnormal color Doppler signals within nerve fascicles or the epineurium.

This case report illustrates how the enlargement of nerves and changes in echogenicity stand out as significant findings in ultrasound examinations. Within this context, the thickening of the ulnar nerve is observed, surpassing a CSA of 10 mm² as it passes through the elbow. Additionally, a substantial enlargement of the C5, C6, and C7 roots is observed (with cross-sectional areas of 15.2 mm², 21.5 mm², and 18.7 mm², respectively), surpassing reference values. These findings should be taken into consideration when evaluating patients with suspected leprosy neuropathy. The asymmetry of CSA when comparing the ulnar nerve with the contralateral limb adds further insight, strengthening the diagnosis of mononeuritis multiplex due to Hansen’s disease, in conjunction with the clinical history and electrodiagnostic results. Mononeuritis multiplex is the most common neurological complication, often affecting the ulnar nerve. It causes motor and sensory impairments that significantly limit hand functionality in severe cases. Therefore, it is essential to achieve early and complementary diagnosis through electrodiagnosis and ultrasonography to improve these patients’ quality of life.
